# Biological consequences of cancer radiotherapy in the context of oral squamous cell carcinoma

**DOI:** 10.1186/s13005-021-00286-y

**Published:** 2021-08-26

**Authors:** G. Feller, R. A. G. Khammissa, M. S. Nemutandani, L. Feller

**Affiliations:** 1 Department of Radiation Oncology, University of Witwatersrand, Johannesburg, Gauteng South Africa; 2 Department of Periodontics and Oral Medicine, School of Dentistry, Faculty of Health Sciences, University of Pretoria, Pretoria, South Africa; 3 Chair of School of Oral Health Sciences, Faculty of Health Sciences, University of Witwatersrand, Johannesburg, South Africa; 4 Office of the Chair of the School of Oral Health Sciences, University of Witwatersrand, Johannesburg, Gauteng South Africa

**Keywords:** Tumour immunity, Immune checkpoint inhibitors, Immunogenic cell killing, Radiotherapy, Ionizing radiation, Bystander effect, Abscopal effect, Oral squamous cell carcininoma, cancer stem cell

## Abstract

Approximately 50% of subjects with cancer have been treated with ionizing radiation (IR) either as a curative, adjuvant, neoadjuvant or as a palliative agent, at some point during the clinical course of their disease. IR kills cancer cells directly by injuring their DNA, and indirectly by inducing immunogenic cell killing mediated by cytotoxic T cells; but it can also induce harmful biological responses to non-irradiated neighbouring cells (bystander effect) and to more distant cells (abscopal effect) outside the primary tumour field of irradiation.

Although IR can upregulate anti-tumour immune reactions, it can also promote an immunosuppressive tumour microenvironment. Consequently, radiotherapy by itself is seldom sufficient to generate an effective long lasting immune response that is capable to control growth of metastasis, recurrence of primary tumours and development of second primary cancers. Therefore, combining radiotherapy with the use of immunoadjuvants such as immune checkpoint inhibitors, can potentiate IR-mediated anti-tumour immune reactions, bringing about a synergic immunogenic cell killing effect.

The purpose of this narrative review is to discuss some aspects of IR-induced biological responses, including factors that contributes to tumour radiosensitivity/radioresistance, immunogenic cell killing, and the abscopal effect.

## Introduction

In the context of cancer radiotherapy, ionizing radiation (IR)-induced biological consequences are observed in targeted cells in the tumour’s field of irradiation that have directly absorbed the deposited IR energy, but survived; in some of their neighbouring cells (bystanders) which have never been directly exposed to the delivered IR; and in the progeny of these cells that were either directly or indirectly exposed to IR effects. These categories of cells usually show genomic instability and are at increased risk of malignant transformation [1–3].

In this scene, the abscopal effect (‘ab-away’; ‘scopus’-target) refers to IR-induced biological reactions occurring distant to the field of irradiation of the primary tumour that are mediated by IR-hit cancer cells and their associated stroma. These biological reactions comprise the rare phenomenon of regression of an untreated distant tumour/metastasis, and the more common one of induction of genomic instability, epigenetic alterations, oncogenic transformation or death of cells, in non-irradiated normal looking tissues [4–7].

Radiotherapy in the context of radiation oncology can be defined as the use of high-energy IR in the form of x-rays, gamma rays, neutrons or protons, in order to kill cancer cells and to control tumour growth. It can be described in terms of dose, fractionation and sequencing, and IR can be used either as a curative, adjuvant, neoadjuvant or as a palliative agent [8, 9]. IR injures cell plasma membrane, nuclear DNA helix and cytoplasmic organelles; alters gene expression and modifies the functional activity of intracellular signalling pathways of both irradiated cells and of non-irradiated bystander cells in the immediate vicinity outside the field of irradiation; and has an impact on the structure and function of the tumour-associated stroma and -microenvironment, particularly on cell-to-cell and cell-to-stroma reciprocal interactions. IR can also modulate anti-tumour immune responses both locally and systemically [5, 10].

IR should be regarded as a ‘double-edged sword’ with the one edge inducing cancer cell death and indirectly augmenting antigen-specific anti-tumour immune responses thus suppressing tumour growth; but the opposite edge has the capacity to initiate and promote oncogenesis by dysregulating cell proliferation, differentiation, migration and survival pathways, and by suppressing local non-specific immune responses [2, 11–14].

The beneficial effects of radiotherapy are mediated by IR-induced irreparable DNA damage which triggers cellular processes of apoptosis, necrosis, mitotic catastrophe or senescence, and by indirectly stimulating anti-tumour immune responses, both resulting in death of cancer cells with consequent control of tumour growth [4, 12, 15, 16]. However, some IR-induced damaged DNA can be efficiently and effectively reconstructed by the cell’s DNA-damage repair mechanisms so that the adaptive abnormal functional activities of the irradiated cancer cells are partially or fully restored. In such cases the tumour cells survive and the tumour may become resistant to further radiotherapy (radioresistance) [2].

IR-mediated oncogenic effects also include the stimulation of immunoinflammatory cells in the tumour microenvironment to release growth factors, cytokines and other biological mediators that can promote genetic instability, angiogenesis, and cancer cell proliferation and invasion [2, 3]. IR has the capacity to trigger in IR-hit cells transcription factors such as nuclear factor kappa β (NF-kβ), activator protein-1 (AP-1), signal transducer and activator of transcription-3 (STAT-3) that upregulate the expression of genes common both to synthesis of inflammatory mediators and upregulation of cell proliferation and survival. Some of these inflammatory mediators can themselves induce cell proliferation and prolonged survival thus facilitating malignant transformation. In turn, the newly transformed precancer and cancer cells have the capacity to synthesize and release proinflammatory and oncogenic mediators, thereby creating a vicious cycle of oncogenic events [17–19]. IR can also induce damage to normal local immunocytes that results in functional impairment of natural killer (NK) and of T cells-mediated protective immune responses, thus enabling cancer cells to evade immune surveillance and to escape immune-mediated killing [2, 3].

Furthermore, during radiotherapy, some collateral IR-induced DNA-damage to bystander cells, and to other non-irradiated cells of normal tissues outside the field of irradiation is unavoidable. These cells with damaged DNA in apparently normal looking tissues may become cytogenetically unstable, thereby having an increased risk of malignant transformation. A minority of persons treated by radiotherapy may acquire over time additional cytogenetic changes that may be induced by any random exogenous or endogenous oncogenic event or agent, and develop second primary cancers, many years later [4, 20].

As effective radiotherapy causes unavoidable toxic side-effects (Table 1), a satisfactory compromise must be found between the IR program which will give the best anti-cancer treatment benefits, and the maximal IR-induced toxicity that can be tolerated [10, 21–24]. Both the risk of developing IR-induced severe toxic side-effects, and the potential for radiocurability varies from person to person and is influenced by age, sex, race, ethnicity, health status, nutrition, body mass and drug interactions; by genetic polymorphism of genes encoding proteins involved in DNA-damage repair and free radical scavenging; by exposure to exogenous radiomodifiers; by certain interactions between polymorphic gene variants and environmental factors; and by the type of the irradiated tissue/organ [9, 10, 20, 21]. As radiobiological responses are complex, adaptive and dynamic, radiotherapy may over time bring about various clinical outcomes comprising cure, partial improvement, no clinical benefits, increased tumour aggressiveness, or a combination of the above [25].

**Table 1:** Early and late IR-induced tissue toxicity

**EARLY EFFECTS** ◦ Skin erythema ◦ Dry or moist skin desquamation ◦ Mucositis ◦ Nausea and diarrhoea ◦ Xerostomia ◦ Osteoradionecrosis	**LATE EFFECTS** ◦ Fibrosis ◦ Atrophy ◦ Neural damage ◦ Second malignancies ◦ Reduced organ functioning ◦ Xerostomia ◦ Osteoradionecrosis ◦ Dysphagia

Legend: Early toxicity appears within a few weeks and late toxicity within months to years from completion of the course of IR. In general, early effects are transient and resolve within a few weeks but late effects are often irreversible and are progressive in nature. The signs, symptoms and functional impairment of IR-induced toxicity is a function of many interconnecting factors including the tissue/organs affected, the specific program of the delivered IR and one’s general health and genetic make-up. Early and late toxicity limits the potency of IR which can be safely delivered, but still be curative [10, 21–24]. Optimizing IR dose distribution, radiosensitizing cancer cells and radioprotecting adjacent normal tissues may go a long way in limiting IR-induced toxicity. Thus, the use of radiomodulators that selectively either increase protection of normal tissues against, or increase sensitization of cancer cells to IR, will allow the safe use of higher dose of IR, which may increase the prospects of radiocurability [9, 21].

Radiotherapy is highly cost effective but radiotoxicity prevents maximising IR doses to the extent that can induce radiocurability without causing unacceptable tissue damage [21, 24]. Therefore, in order to improve the therapeutic index of radiotherapy, the efficacy of IR-induced cell killing and/or the level of radioprotection of normal tissues must be enhanced. The use of radiosensitizers that target cellular DNA damage response, cell-cycle checkpoints, cell-to-cell and cell to extracellular matrix (ECM) signalling pathways, oncometabolites, tumour microenvironmental elements (i.e. vascularisation, oxygenation) and immune checkpoint regulators can promote radioresponsiveness and may result in increased IR-induced cell killing; and the use of ‘radioprotectors’ such as free radical scavengers, inhibitors of certain IR-induced intracellular transduction pathways and agonist of relevant Toll-like receptors may selectively protect normal tissues from toxicity while maintaining IR-induced efficacy of cancer cell kill [13].

The purpose of this narrative review is to increase the mechanistic understanding of IR-induced pathobiological tissue responses and immunogenic cell killing in the context of oral squamous cell carcinoma. Data for this review was obtained by searches of MEDLINE and PubMed using the search terms tumour immunity, immunogenic cell killing, cancer radiotherapy, bystander effect, abscopal effect, cancer stem cell, oral squamous cell carcinoma; and of references from relevant articles that were deemed pertinent. English-language academic papers, but not those published in a language other than English were scrutinized for the writing of this review.

## Oral squamous cell carcinoma

Oral squamous cell carcinoma (SCC) is a malignancy of genetically altered oral epithelial cells (keratinocytes) which are characterized by having the capacity of uncontrolled proliferation and increased survival, and by the capacity to invade the underlying connective tissue through the basement membrane and subsequently cause destruction of local tissues. This malignant phenotype is driven by gene-environment interactions that bring about abnormal activation of certain intracellular transduction pathways which in turn trigger relevant downstream transcription factors that regulate expression of genes that mediate cell attachment, proliferation, differentiation, migration and apoptosis; by random genetic mutations; and by intrinsic non-genetic microenvironmental factors such as inflammation and altered mechanical properties of extracellular matrix [26].

Oral SCC is the most common malignancy of the oral cavity accounting for 90% of all oral cancers and is predominantly a disease of elderly males, though its incidence is increasing significantly among the younger-age population groups [23, 27, 28]. Oral SCC may affect any oral mucosal site but most commonly the tongue, followed by the floor of mouth, the gingiva and alveolar mucosa. The main risk factors for the disease include a long history of tobacco/betel/areca nut use, of drinking alcoholic beverages and of a diet poor in fresh fruit and vegetables [27, 29]; and subjects with oral SCC have a relatively low survival rate. Failure of treatment manifests in a loco-regional recurrence or distant metastasis, that occur in 30–50% of cases [29, 30].

The incidence and prevalence and other epidemiological variables related to oral SCC differ greatly among different populations living in different geographic locations of the world, and among different ethnic groups within the same population. This variability is probably related to genetic factors, to environmental-specific factors and to exposure to ethnic-specific high-risk habits and life styles [27]. As no screening strategy has been found to be effective for early diagnosis, attentive physical examinations remain the central approach for early detection of oral SCC [30].

Most oral SCC develop within fields of precancerized oral epithelium by clonal expansion of a genetically altered stem/progenitor cell in the basal cell layer of the oral epithelium, forming fields of precancerized epithelium which contain keratinocytes at different molecular stages of transformation [31]. These precancerized fields of oral epithelium may appear clinically normal or may manifest as potentially malignant lesions such as erythroplakia or leukoplakia. It is estimated that up to 35% of oral SCCs emerge from pre-existing potentially malignant lesions while the remainder arise *de-novo* from apparently normal looking precancerized epithelium containing genetically altered keratinocytes [29].

Clinically, oral SCC may present as leukoplakia or as erythroplakia, as a necrotic ulcer with irregular raised indurated borders, as a broad based exophytic mass with a surface texture that can be relatively smooth, verrucous or pebbled, or as a combination of the above [29]. The clinical course of oral SCC is unpredictable and its management requires the services of a multidisciplinary team. The selection of a specific treatment option is dictated by patient-related factors including age, general health, anticipated functional and cosmetic outcomes, financial cost, high-risk habits/life-style; and by carcinoma-related factors such as tumour-node-metastasis (TNM) staging system and histopathological features [22, 32].

Histopathological determinants include grade of differentiation, growth pattern of invasion, depth of invasion, vascular/neural invasion, bone involvement and nodal status (number of lymph nodes involved, size of largest metastasis, extracapsular spread, extranodal extension). It appears that the subtype of tumour infiltrating lymphocytes and the number of antigen-presenting dendritic cells in the immunoinflammatory cell infiltrate, as detected by immunohistochemistry studies may influence the decision taken regarding the preferred treatment modality [28].

Treatment options for oral SCC include excision/resection, radiotherapy, radio-immunotherapy, systemic cytotoxic chemotherapy, target therapy or various combinations of these modalities, either concurrently or in an orderly sequence. Whenever possible surgery is the preferred first line of treatment, but in advance stage or recurrent oral SCC, a combined treatment program is obligatory [24, 32]. Radiotherapy is practiced as a primary line of treatment for patients with unresectable tumours or those who cannot undergo surgery; as an adjunct to primary surgery for advanced stage tumours and when the tumours histopathological features constitute high-risk for recurrence, and in such cases for the best clinical outcomes IR should be delivered within 6 weeks from surgery; as a salvage treatment in persistent or recurrent disease; or as a palliative treatment [23, 33, 34]. Elective radiation of draining lymphnodes related to the primary tumour is controversial since in these lymphnodes, dedicated antigen-presenting dendritic cells cross-prime cytotoxic T cells with subsequent generation of tumour antigen-specific cytotoxic T cell responses, thereby IR may interfere with immunogenic killing of cancer cells [14].

A total of 60–70 Gy of IR delivered to the primary tumour and associated involved lymphnodes constitute the conventional protocol of radiotherapy for the treatment of oral SCC but this should be tailored according to patient-and-tumour specific factors. In order to reduce IR-induced toxicity, the IR dose is fractionated into smaller doses, usually delivered in 1.5–2.25 Gy per fraction on daily basis [14, 18, 24]. Importantly, IR doses exceeding 72 Gy typically provoke unacceptable toxicity [18].

## Ionizing radiation, epithelial field of precancerisation and secondary primary tumours in relation to oral SCC

IR may induce the de novo evolution of an epithelial field of precancerization *via* bystander out-of-field mechanisms described above; or may promote malignant transformation of already genetically altered keratinocytes within pre-existing apparently normal looking epithelial fields of precancerisation [35, 36]. Fibroblasts in the oral SCCs-associated stroma are also likely to undergo cytogenetic and epigenetic alterations, induced by the same genotoxic and mutagenic agents that mediated the transformation of the keratinocytes in the overlying epithelial field of precancerization. These carcinoma-associated fibroblasts within the carcinoma-associated stroma have the capacity to further promote carcinomatous transformation and tumour growth, and conversely the carcinomatous cells can induce biological changes in the local microenvironment that support tumour growth and invasiveness [36].

Ionizing radiation may alter the tumour microenvironment by affecting the reciprocal communications between cells and between cells and their extracellular matrix (ECM), and by influencing cell metabolism and cell phenotype, all of which can promote tumourigenesis. For example, fibroblasts in the irradiated stroma can secrete tumour growth factor β1 (TGF-β1) and reactive oxygen species (ROS) that have an impact on the phenotype of keratinocytes, on the process of epithelial-mesenchymal transition (EMT) and on the function of local immunocytes; and can secrete matrix metalloproteinases 9 (MMP 9) and beta fibroblast growth factor (bFGF) which can mediate vascular remodelling and influence the composition of the tumour inflammatory cell infiltrate. Taken together, these functional activities of carcinoma-associated fibroblasts promote cell transformation and tumour growth [37, 38].

The genetically altered keratinocytes in the field of precancerization may have identical molecular profiles if they evolved from one stem/progenitor basal cell that has underwent clonal expansion to form a monoclone; may have similar but not identical molecular profiles if they descended from subclones that evolved by clonal divergence from a monoclone, hence being clonally related; or may have dissimilar molecular profiles if they evolved from several stem/progenitor basal cells that underwent an independent initial transformation and subsequent unrelated clonal expansion, giving rise to a polyclonal population of precancerised keratinocytes [20, 39]. As IR or any other cancer therapy cannot eradicate the entire field of precancerization, because it is neither clinically or histologically detectable, nor its extent determinable, persons who have been treated for primary oral SCC with IR are at increased risk of developing a ‘new’ cancer in the field of precancerization [20].

Thus, the development of a new oral SCC in the immediate or general vicinity of the primary oral SCC, sometime after apparently successful treatment of the primary tumour, may be a loco-regional recurrence if the two carcinomas show identical genetic profiles; may be a new field carcinoma that evolved from a subclone of cytogenetically altered cells within the same field of precancerization if the genetic profiles of the two carcinomas are similar but not identical; or may be a second primary tumour arising from a different clone of cytogenetically altered cells in the same field of precancerization if the genetic profile of the two carcinomas are dissimilar [32].

## Cancer stem cells in relation to oral SCC and to ionizing radiation

A small minority of cells within an oral squamous cell carcinoma, one-to-five percent, comprise tissue-specific cancer stem cells (CSCs), which function as cancer precursor cells (cancer-initiating cells) [40]. As in the case of their normal counterparts, CSCs have an undifferentiated phenotype but possess the potential for further differentiation; have a limited proliferative capacity; and have a relatively unlimited self-renewal capabilities. CSCs contain the genetic information of the tissue, maintain active expression of teleromase and are slow-cycling and resistant to apoptotic signals [25, 41–44].

Tissue-specific stem cells may have undergone cytogenetic and epigenetic alterations and consequently have acquired a CSC phenotype during any stage of embryogenesis or post-developmentally. Alternatively, it is possible that CSC originate either from a transient-amplifying cell or from a post-mitotic cancer cell that has undergone cytogenetic alterations culminating in de-differentiation into an analogue of an immature CSC expressing some developmental transcription factors and intracellular signalling pathways characteristic to a CSC phenotype [41, 44].

However, it is not clear whether such analogues have also acquired self-renewal properties to initiate and sustain cancer growth [41, 44]. In any event, division of a CSC, as in the case of their normal counterpart, gives rise to two daughter CSCs which remain in the designated stem cell niche or to one CSC which remains in the stem cell niche, and to a second daughter transient-amplifying cell that leaves the stem cell niche but remains in the progenitor cell compartment in the basal and suprabasal layers of the epithelium. Subsequently, the transient-amplifying cells in the progenitor compartment undergo mitosis and give rise to two daughter transient-amplifying cells which remain in the progenitor cell compartment, and to two daughter cells which exit the progenitor cell compartment and begin to differentiate and enter the maturation process gradually rising to the surface of the epithelium as post-mitotic keratinocytes [41–43].

The vast majority of cells within a carcinoma are transient-amplifying cancer cells and post-mitotic cancer cells. The transient-amplifying cancer cells have increased survival, extended proliferative potential, and an aggressive biological behaviour, all of which confer upon them the capacity to invade and destroy local tissues; on the other hand, post-mitotic cancer cells are at different stages of differentiation and maturation, having very little or no proliferative potential. Since neither transient-amplifying cells nor the post-mitotic cells have a self-renewal capacity, it is unlikely that this large proportion of tumour cells can initiate either metastatic or second primary tumours, or can sustain tumour progression and growth [44, 45]. Of significance, it appears that IR may induce irradiated non-CSCs to de-differentiate, acquiring a genetic program characteristic to CSCs, thereby enlarging the pool of the CSC population [25].

Thus, a carcinoma consists of cytogenetically-altered keratinocytes at different stages of malignant transformation [41, 46] and this heterogenous cell population comprises CSCs, transient-amplifying cells, and post-mitotic carcinomatous cells, but only cells with a CSC genotype can initiate, drive and sustain the uncontrolled proliferation of primary and metastatic cancerous cells [25, 41].

It appears that in comparison to non-CSC within the tumour, CSCs are more resistant to biological consequences induced by direct hit of IR, and by bystander-related mechanisms [2]. This is probably owing to increased expression of genes encoding anti-apoptotic and DNA repair proteins [40]. Thus, as CSCs are more radioresistant than non-CSCs, the reduction in tumour mass following IR is predominantly owing to the death of non-CSCs, but not of the CSC subpopulation [9, 44]. It is probable, that even after IR-induced complete clinical resolution of the tumour, local recurrence sometime later, is owing to one or several CSCs which have survived the radiotherapy and maintained their oncogenic cellular stemness, and which, by clonal expansion give rise, again to a monoclonal cancer cell population, thereby initiating and sustaining growth of the recurrent cancer. Therefore, the clinical observation of IR-mediated tumour resolution should not be the sole criteria for acknowledging radiocurability [43, 44].

## Some ionizing radiation-induced biological consequences of cancer radiotherapy

Tumour-related clinical, anatomical, histopathological and molecular factors, as well as the tolerance limits of the surrounding normal tissues outside the field of irradiation to IR-induced toxicity, dictate the nature of the radiation treatment program (IR dose, fractionation, sequencing and duration) and influence the outcome of the radiation therapy [44].

IR induces cell death directly by causing irreparable damage to nuclear DNA double helix, or indirectly by ionizing cellular water with the generation of reactive oxygen species (ROS) and other free radicals which then damage the DNA chains, and vital cellular components essential for cell functioning [2, 47]. Furthermore, IR induces exposure, release and spread of tumour neo-antigens (antigen spread/antigen cascade) from the field of irradiation of the primary tumour which subsequently boost immunogenic killing of tumour cells [48].

IR can also lead to the release into the local microenvironment of biological agents which have the capability to induce chromosomal damage and genomic instability in cells outside the field of irradiation (out-of-field), and of cytokines, growth factors and inflammatory mediators which favour tumour growth. Sometimes, IR may also promote local invasion and induce seeding of surviving viable tumour cells from the irradiated field into the circulation with the risk of developing a metastatic growth [4, 8, 49–52]. However, in general, the beneficial effects of IR overshadow the negative ones [11].

IR has the capacity to induce additional cytogenetic alterations upon some irradiated primary tumour cells, which may then genetically evolve, acquiring a complete set of genetic alterations of a metastatic phenotype. This metastatic genetic profile may enable the affected cells to negotiate the physical and immunological obstacles of leaving the primary tumour, of invading blood and lymphatic vessels, of survival in the circulation during transport to a distant site, and of establishing micrometastasis and subsequent metastatic growth [45, 53].

These properties of primary tumour cells with a metastatic phenotype, some of which may have been facilitated by IR, are mediated by functionally dysregulated intracellular transduction pathways and by altered gene expression. Aberrant interactions between stromal cells and cancer cells, and humoral factors including cytokines, chemokines and growth factors may play a role in influencing tropism of metastatic cells for specific distant tissues and may promote metastatic colonization and infiltration [54].

In this context, radiation induced DNA damage can trigger NF-κB intracellular signalling pathways with consequent increased expression of downstream effector proteins that mediate cell proliferation, pro-survival and anti-apoptotic processes, and radioresistance. Some of these proteins including cyclin D1, B-cell lymphoma 2 (Bcl-2), tumour necrosis factor α (TNF-α), vascular endothelial growth factor (VEGF), X-linked inhibitor apoptosis protein (XLAP), matrix metalloproteinase 9 (MMP 9) and cyclooxygenase 2 (COX-2) play an important role in determining the tumour biological behaviour and consequently the clinical course of the disease [18].

Some IR-induced cellular metabolites termed oncometabolites are essential to the oncogenic process as they can dysregulate anti-tumour immune responses, expression of genes relevant to oncogenesis, and intracellular signalling pathways that mediate repair of damaged DNA; can promote tumour’s hypoxic and inflammatory phenotypes; and can impact on the tumour’s anti-oxidant capacity and its radioresistance/radiosensitivity properties. Thus, the functional activity of IR-induced oncometabolites play an important role in determining the outcome of radiotherapy [55].

## Ionizing radiation as it relates to tumour microenvironment

IR-induced changes to the cellular and extracellular matrix (ECM) structures in the field of irradiation can disrupt integrin expression and function, assembly of focal adhesion proteins, cytoskeletal architecture, cell-to-cell and cell-to-ECM communications and adhesions, all of which may contribute to IR-mediated oncogenesis [26] and determine tissue radioresponsiveness [56]. IR can stimulate the intracellular TGF-β1 signalling pathways with consequent upregulation of expression of TGF-β1 target genes encoding some ECM proteins. For example, the expression of basement membrane fibrillar proteins and collagens, and of enzymes involved in ECM remodelling are enhanced post-radiation and play an important role in mediating IR-induced fibrosis and loss of function [57].

Physiologically, the intracellular molecular transduction pathways form a network which constitute a complex dynamic adaptive system, with each pathway being influenced by other pathways, and the aggregate of the integrated activity of several interacting pathways mediate the expression of relevant genes. In turn, the cross-talk between relevant genes and their organisation within gene regulatory networks together with the outcome of the interaction between different intracellular signalling pathways will determine the normal structure and function of the cell [26].

Genotoxic environmental agents (tobacco, alcohol, IR), microenvironmental biological agents with potential oncogenic activities (certain cytokines and growth factors), and mutations in certain essential regulatory genes, can induce alterations in the functional activity of intracellular transduction pathways and in the profile of the gene networks which may lead to malignant transformation and cancer development on the one hand [26], and influence radioresponsiveness on the other hand.

## Hypoxia and heat shock proteins in relation to cancer radiotherapy

Compared to normal cells, rapidly proliferating cancer cells require more oxygen, glucose and amino acids for their functional activity and this may lead to the development of a hypoxic tumour microenvironment [9, 58]; and since the availability of molecular oxygen (O_2_) is critical to formation of IR-induced reactive oxygen species (ROS) and consequent cell death, a hypoxic tumour microenvironment that is characteristic to many advanced primary tumours, confer resistance to radiotherapy [9, 25]. Furthermore, under tumour microenvironmental conditions of O_2_ deficiency, some oncometabolites may upregulate the expression of hypoxia-inducible factor 1 (HIF-1) which in turn can re-program the cell’s metabolic activities in order to balance the deficiency in O_2_ [55].

HIF-1 is a heterodimeric transcription factor (HIF 1α/HIF 1β) that controls expression of genes that regulate oxygen homeostasis and responses to environmental stressors including IR. IR-induced production of ROS and damage to nuclear DNA can activate HIF 1α and its target genes, some of which encode angiogenic growth agents and antioxidants that reduce the efficacy of IR-induced cell killing, thereby enhancing radioresistance [58]. Furthermore, hypoxia can promote genetic instability, intratumoural heterogeneity and inhibit local immune responses all of which enhance tumurogenesis and radioresistance [59].

Heat shock proteins (Hsp) are evolutionary highly conserve proteins that are produced and released in response to cellular stress. Hsp 70 and Hsp 90 are overexpressed by tumour cells, and contribute to cell proliferation, invasiveness and metastasis, and also hinder senescence and apoptosis, thus promoting tumour growth. However, on the other hand Hsp are also powerful stimulators of innate immune reactions, hence facilitating immunogenic cell killing [60, 61].

Membrane-bound Hsp 70 expressed by tumour cells, and extracellular membrane-free Hsp 70 found in the tumour microenvironment can activate NK cells and antigen-presenting dendritic cells, and can mediate the release of certain immunoinflammatory cytokines, all of which enhance anti-tumour immunity. Hsp 70 and Hsp 90 act as ‘danger-associated molecular pattern’ agents that can bind to and trigger the Toll-like receptor 5 (TLR-5) and TLR-4 on antigen-presenting cells to produce cytokines that activate and enable NK cells (and possibly cytotoxic T cells) to launch a cytoloytic attack against Hsp-positive tumour cells. As IR enhances the expression of Hsp 70 and Hsp 90 in the tumour microenvironment, IR-induced upregulation of Hsp may promote immunogenic killing of tumour cells [60, 61].

## Mechanisms of ionizing radiation-induced bystander effect

The size of the irradiation field of the targeted tumour is substantially larger than the margins of its gross volume as calculated from clinical examination and imaging studies. This is because the actual size of the irradiation field must also accommodate for additional neighbouring tissue surfaces that may harbour subclinical disease, and for errors related to patient positioning, target location and instrument accuracy that are not uncommonly made in routine clinical practice by radiation-oncologists. This inclusion of additional normal tissue into the irradiated field is not without clinical consequences since it exaggerates IR-induced toxicity and inflammatory reactions, and may reduce the efficacy of immunogenic cell killing in the tumour microenvironment [14].

In the context of cancer radiotherapy, IR-mediated bystander effect refers to the structural and functional changes observed in cells which border the field of irradiation of the primary tumour, and which have not been directly exposed to IR (out-of-field effect). These biological effects comprise genomic lesions including chromosomal damage, gene mutation, sister chromatoid changes epigenetic alterations and formation of micronuclei, and are caused by irradiated cells in the field of irradiation that have directly absorbed the delivered IR energy, but survived; and by extracellular irradiated targets in the microenvironment of the field of irradiation [1, 3, 62, 63]. Out-of-field bystander effects are mediated by signals transmitted through gap junctional intercellular communication, and by secreted diffusible signalling molecules, which drive communications from cell-to-cell across the ECM [16, 62].

Cancer cells within the irradiation field and volume, which have been directly hit by IR but survived, can reciprocally cross-talk, and interact with un-hit cancer cells within the same irradiated field/volume, to generate cellular responses by the same mechanisms explained above that drive the bystander effect. The biological consequence is amplification of IR-induced tissue damage and cell death [2].

Gap junctional intercellular communications that allow passage of free radicals, ca^2+^ ions, nucleotides and peptides, together with soluble factors released from irradiated hit cells, including TNF-α, TGF-β, interleukin (IL)-6, IL-8, IL-1β as well as ROS, reactive nitrogen species (RNS), arachidonic acid metabolites and lipid peroxidation products are essential for the generation of the bystander effect. These signalling agents can trigger intracellular transduction pathways in bystander cells that bring about genomic instability with subsequent increased cancer risk [1, 2, 8, 47, 50].

## Ionizing radiation induced anti-tumour immune responses

Owing to intrinsic factors such as genomic instability, mutations and clonal evolution, and as a response to external selective pressures imposed by the immune system, tumour associated antigens (TAA) undergo continual immunoediting. This may lead to loss of immunogenic peptides, thus allowing tumour cells to evade the immune surveillance and then to escape immunogenic cell killing; but conversely, it may also lead to the generation of immunogenic TAA (neo-antigens) that have the potential to trigger antigen-specific protective T-cell responses [7, 12, 16].

The actual existence of the tumour is evidence to the failure of the immune system to prevent its development, and later its progression. Tumour cells can escape immunogenic cell killing by releasing immunosuppressive cytokines, by expressing cell surface molecules that inhibit T-cell functions, and by recruiting to the tumour microenvironment immunosuppressive M2-phenotype macrophages, myeloid-derived suppressor cells and regulatory T cells. All these confer upon the tumour an immunosuppressive and telerogenic microenvironment [12, 16, 64].

Tumour cells that survive IR-induced direct killing, may have undergone genotypic and phenotypic alterations that include increased expression of death receptors, MHC class 1 molecules, co-stimulatory molecules, adhesion molecules and stress/danger ligands, all of which increase tumour cell susceptibility to immunogenic killing [4, 12–14, 19, 40, 65].

Furthermore, IR-induced damage to tumour cells exposes and releases TAA, some of which are immunogenic and therefore can be recognized, captured and processed by immature antigen-presenting-dendritic cells. As they migrate to draining lymphnodes, the dendritic cells undergo a process of maturation, which enable them to efficiently present the tumour antigens to naive T cells, cross-prime them, and induce antigen-specific T cell immune responses [11, 12, 17]. The activated effector, antigen-specific cytotoxic T cells lyse immunogenic tumour cells with subsequent release of additional TAA that further boost anti-tumour immune responses [66].

In general, the efficiency and efficacy of the cytotoxic T cell reactions generated towards TAA are determined by the co-stimulatory receptors expressed by the antigen-presenting cells, by the type of pattern-recognition receptor triggered, by the nature of the molecular structure of the antigen and by the type of the biological mediators released in the tumour microenvironment [67]. Danger signals generated by tumour cells and by IR-induced tissue damage including adenosine triphosphate (ATP), high mobility group protein 1 (HMGP1), calreticulin, hypoxia, heat shock proteins and reactive oxygen/nitrous metabolites play an important role in determining the magnitude and rapidity of the generated immune response [14, 67, 68].

Thus, it appears that by augmenting the expression and release of TAA in the local microenvironment, IR turns the primary tumour into a personalized in-situ vaccine that has the capacity to stimulate antigen-specific T cell responses [12, 50, 64, 66, 69]. These activated lymphocytes, when enter the circulation, may protect against metastatic spread and against early local recurrence; and may explain the rare occurrence of regression of untreated metastasis at a distance, outside the field of irradiation of the targeted primary tumour (abscopal effect) [5, 9, 12, 70, 71].

In the rare instances that it occurs, the IR-mediated beneficial abscopal response does not materialize concurrently with the delivered IR to the primary tumour but rather occurs later, over a period of months beyond the conclusion of the IR treatment. This suggests that to mount a robust anti-metastasis immune response requires both an inherent competent immune system, and sufficient time to construct an antigen-specific cytotoxic T cell reaction that can efficiently and effectively operate distant to the irradiated primary tumour. In such cases, the primary tumour functions as an ‘immunogenic reactor’ [69].

However, on the other hand, IR can mediate inflammatory reactions, oncogenic processes and even cell killing distal to the irradiated field/volume of the primary tumour, causing damage to normal tissue that may be clinically significant. This harmful process is driven by signalling molecules such as proinflammatory cytokines, oncogenic mediators and tissue damage-associated molecular patterns that are released from the field of irradiation and enter the circulation triggering systemic inflammatory reactions that may generate a detrimental abscopal effect [3].

## Radioresponsiveness (radiosensitivity/radioresistance) in relation to radiocurability

The functional activity of intracellular signalling pathways of cancer cells that regulate biological tasks such as repair of damaged DNA, apoptosis, cell cycle progression, angiogenesis and immunoinflammatory reactions determine the radiosensitivity/radioresistance properties of the tumour. For example, downregulation of inhibitory damaged-DNA repair and of apoptotic signalling pathways, hypoxia, increased capacity of scavenging of reactive oxygen species, great number of stem cells, tumour cell heterogeneity and disrupted vasculature, all enhance radioresistence and thereby reduce the prospects of radiocurability [2, 7, 72].

Radioresponsiveness, to a major extent, is determined by the quantum of the IR-induced DNA damage; and by the functional capacity of the DNA-damage repair mechanisms of the irradiated tumour cells which dictates whether the damage is reparable and the hit tumour cells survive, or whether it is irreparable, leading to cell death via apoptosis, necrosis, sensescence, mitotic catastrophe or authophagy [40].

The physiological functioning of p53 and epidermal growth factor receptor (EGFR) intracellular signalling pathways are critical for cellular DNA-damage response (DDR)-mediated repair of damaged DNA; but in oral SCC the expression of p53 mutations and of EGFR gene are upregulated so that the capacity of repair of damaged DNA is enhanced leading to increased longevity of tumour cells, and tumour resistance [40]. In this context, both gain-and loss-of-function mutations of p53, regardless the mechanisms involved, may promote damaged DNA repair and cell survival and inhibit pro-apoptotic pathways; and hyperactivation of EGFR intracellular signalling pathways facilitates prolonged cell survival and proliferation. All these can enhance the radioresistent properties of the tumour, with increased likelihood of IR treatment failure [40].

Several intracellular survival signalling pathways can be activated by IR. These include phosphoinositide 3 kinase (PI3K), activated protein kinase (AKT), mitogen activated protein kinase (MAPK), nuclear factor-κB (NF-κB), mammalian target of rapamycin (mTOR) and TGF-β pathways. These play a crucial role in regulating mechanisms of DNA-damage repair and of cell death which influence the radioresponsiveness properties of the tumour. Consequent to tumour radioresistance, and to IR-induced toxicity which limits the delivery of high doses of IR necessary for achieving radiocurability, in most cancer cases, radiotherapy should be administered in combination with other anti-cancer treatment modalities such as immune response modifiers [51, 73].

## Immune response modifiers

Immune response modifiers also termed biological response modifiers or immunoadjuvants are agents that boost immune responses, and in the context of cancer treatment include tumour vaccines, cytokines, interleukins, colony stimulating factors, stem cell growth factors, monoclonal antibodies which target inhibitory immune checkpoint receptors and inhibitors of tumour necrosis factor [7].

The delivery of IR together with the use of immunoadjuvants as a combined anti-tumour treatment modality, compared to monomodal treatment of either radiotherapy or immunotherapy alone, has a synergistic immunogenic cell killing effect. This is because IR upregulates the expression of TAA thus providing to effector immunocytes additional immunogenic targets to attack, while immunoadjuvants boost the competency of anti-tumour immune responses. Such a combined treatment modality, depending on the type of the immunoadjuvant used, can upregulate dendritic cell maturation and functional activity, cross-priming of effector T cells, the diversity of T cell receptor repertoire, trafficking of lymphocytes and T cell mediated immune responses. All these, may ameliorate the intrinsic immunosuppressive properties of the tumour microenvironment thereby facilitating immunogenic cell killing [6, 11, 16].

The inhibitory immune checkpoint regulators, the cytotoxic T lymphocyte-associated antigen 4 (CTLA-4) and the programmed cell death-1 (PD-1) are T cell receptors which transduce inhibitory signals thereby downregulating T cell activity [14, 16]. While the immune checkpoint CTLA-4 interferes with T cell cross-priming by dedicated antigen-presenting cells in the draining lymphnodes thus reducing the available number of activated T cells, inhibitors of the checkpoint PD-1 interfere with the functional activity of already primed/stimulated T cells in the tumour microenvironment [16]. Under physiological conditions these negative immune regulators are critical for dampening hyperactive immune responses, but in the context of cancer biology, the activation of inhibitory immune checkpoint receptors by TAA, downregulates immunogenic killing of tumour cells. Blocking the activity of these inhibitory checkpoint receptors therefore, may potentiate anti-tumour immunity and consequently improve clinical outcomes [11, 14, 64].

For example, pilimumab is a monoclonal antibody that blocks CTLA-4 receptor and pembrolizumab is an antibody that targets the PD-1 or its ligand PDL-1. Combination of radiotherapy and immune checkpoint inhibitors can boost immunological killing of tumour cells, and can increase the probability of a beneficial abscopal eventuality [16, 49, 64]. A similar synergistic anti-tumour immunogenic effect can be achieved by combination of radiotherapy and granulocyte-macrophage colony stimulating factor (GM-CSF). GM-CSF is a cytokine with a growth factor activity that upregulates mobilization, differentiation, and function of antigen-presenting dendritic cells, so that dendritic cell cross-priming of effector T cells is enhanced, resulting in the potentiation of immunogenic cell killing [6, 7, 12, 50].

## Conclusion

Most subjects with oral SCC present with an advance stage disease at the time of diagnosis and are usually managed with a combined treatment modality often incorporating radiotherapy. In addition to directly inducing cancer cell death, IR-mediated damage to tumour microenvironment brings about exposure and release of tumour neoantigens that can be recognised and attacked by the immune system. The combined use of IR and immune checkpoint inhibitors synergize immunogenic cell killing locally at the irradiated primary tumour site, and rarely also at metastatic sites, outside the field of irradiation.

A better understanding of the impact that IR has on the bidirectional cell-to-cell, cell-to-ECM, and cell-to-stroma interactions is needed in order to identify molecular targets that can be used for radiosensitization with the view of improving the rates of radiocurability.

## Data Availability

Not applicable.
